# Book Reviews

**Published:** 1913-08

**Authors:** 


### BOOK REVIEWS.

Selected Papers of Dr. Carlos J. Finlay, published by the Secretary of Sanitation and Benevolence of Cuba. Dr. Manuel Varona Suarez. 657 pages, illustrated, alternately in English and Spanish.

This publication was originally decreed by the Provisional Government in 1908 and has been published by the present government. Dr. Finlay's work in regard to tropic and semi-tropic diseases is well known, so that this volume cannot be regarded merely as a monument to his work in Cuba, but, especially as it contains previously unpublished matter, it ranks high as a practical and modern medical publication.

Publications of the Massachusetts General Hospital, Vol. 4, No. 1, January, 1913.

This contains a number of very valuable contributions on various medical and surgical topics. We believe, as a general principle, that matter of so great value to the profession should appear in regular periodicals and not in special publications or transactions.

Proceedings of the Royal Society of Medicine, Vol. 6, No. 6, April, 1913. Published by Longmans, Green & Co., London; 7/6d.

One of the most interesting articles, dealing extensively with statistics, is on "The House as a Contributory Factor in the

Death Rate," by A. K. Chalmers. We are surprised to note that the death rates, while showing a diminution of from 25 per cent. down to 3.6 per cent. for the respective number of apartments, in contrasting statistics of 1901 with the average for 1909-12, are progressively higher as we pass from one apartment to two, three and four-apartment houses. It occurs to us that this is due to a difference in dialect, the English not recognizing the nice distinction between apartment and compartment insisted on in America, so that the term four-apartment does not indicate a building consisting of four flats, as in America, the numbers rather indicating rooms in what we should call apartments, in tenement houses.

---

Transactions of the Fifth International Sanitary Conference of the American Republics, held in Santiago de Chile, November 5-11, 1911. Published and distributed under the auspices of the Pan-American Union, John Barrett, Director-General, Washington, D .C.

This conference dealt with a miscellaneous series of topics, as suggested by the experience of different countries. The repeated collation of such experience will ultimately be of great value, not only as regards international problems, but also those affecting individual countries. The Pan-American Union deals with many political problems and the good work of Mr. Barrett is well known to statesmen and sociologists.

---

The Doctor's Riot. This is a pamphlet containing fac similes of illustrations and items in old newspapers dealing with the riot in New York in 1788, on account of suspected grave robbing for anatomic purposes. This riot, while apparently unjustified, ultimately led to more liberal legislation regarding the supply of anatomic material for medical schools. This pamphlet is issued by Messrs. Reed & Carnrick of Jersey City. The editor has, for some years, preserved in a file of "History and Art of Medicine via Advertisers" similar publications. Such a file becomes of considerable value as it gradually accumulates, and we take pleasure in acknowledging this contribution to medical literature.

---

Transactions of the Fifteenth Annual Meeting of the American Gastro-Enterologic Association at Atlantic City, June 3 and 4, 1912.

This is a neatly bound collection of reprints, covering a wide range of topics, discussions, business proceedings, lists of officers and members, etc., being included.

ANNUAL REPORT OF THE DEPARTMENT OF HEALTH OF BUFFALO for the year 1912.

This deals in detail with the manifold activities of the Department and reflects great credit on the Commissioner, Dr. Francis E. Fronczak, and his staff.

---

COAGULOSE. Pamphlet issued by Parke, Davis & Co. This describes the treatment of haemorrhage with precipitated blood serum, as elaborated by G. H. A. Clowes, Ph. D., and F. C. Busch, M. D., of Buffalo. This method seems destined to revolutionize the control of haemorrhage, short of gross lesion obviously requiring ligation. We feel a sense of local pride that the method is a new achievement for the special territory of this JOURNAL. Both the originators of the method and the firm who have shown so great acumen in placing the discovery on a practical commercial basis, have our congratulations.

---

LOCATION OF GASOLENE ENGINE TROUBLES MADE EASY. By Victor W. Page, M. E., published by the Norman W. Hanley Publishing Co., 132 Nassau St., New York; 25 cents.

This is a chart with a large diagram of a typic automobile engine, following the analogy of a table of differential diagnosis familiar to the medical profession. The table is arranged by cardinal symptoms, lost power and over-heating, noisy operation, skipping. Under these general headings the gross anatomic location, pathology, symptomatology and therapeutics are given. With the aid of this table, random vivisections of cars on the street will become less frequent and, with due regard to the considerable differences in comparative anatomy, prompt operation, intelligently directed, will prove of great prophylactic value.

---

THE OPERATING ROOM AND THE PATIENT, by Dr. Russell S. Fowler of Brooklyn; published by the W. B. Saunders Co., Philadelphia and London; 611 pages, 212 illustrations; $3.50; third edition.

This work is a recognition of the growing importance attached to surgical detail, especially in avoiding misfortunes due to imperfect equipment and to disregard of the status of the patient himself. It is particularly valuable to the occasional operator and to the one forced to operate in emergencies and without the help of a fully equipped and well manned operating room.

METHODS AND MORALS IN MEDICAL PRACTICE, or The Evolution of the Bath Robe. By A. J. Whitworth, M. D.

This is a red covered pamphlet, including the certificate of an attorney that it is fit to pass through the mails, and it contains an autobiography stating the author's qualifications for judging of erotic matters, which include actual experience as an osteopath. It is a scathing denunciation of the "Knight of the Hand and Robe" and, while we trust that the serious sexual results charged against Osteopathy, are exaggerated, and wish that the manuscript might have been revised before publication, this book certainly calls attention to a matter deserving the consideration of the profession and of sociologists. We have, personally, ever since the development of osteopathic practice, stated bluntly to women patients asking advice regarding such manipulations, that we did not consider it decent for a woman to subject herself to manipulation by a man, unless under certain circumstances in which a masseuse was not competent, and unless proper chaperonage was provided. And we quite agree with the author that repeated and protracted manual rubbing of a practically nude female by a practically nude man presents no true analogy to the necessary, brief and occasional exposure of a patient by a regular physician for technical diagnostic and therapeutic proceedures.

---

WHAT HEART PATIENTS SHOULD KNOW AND DO. J. H. Honan, M. D., Bad-Nauheim, Germany; published by Dodd, Mead & Co., New York; 204 pages; $1.20.

This is an intelligent, conservative and well-written book—of its kind. This limitation is a frank confession of personal prejudice toward any semi-popular presentation of systems of hygiene and therapeutics, with information to the patient regarding his own condition. This prejudice is founded on the following conceptions: That no similar work can afford the competent physician information which he does not already possess: that it is impossible to translate into non-technical language, technical scientific points which the technically uneducated persons will always grasp in their true significance; that what the "heart patient should know" or what any other patient should know about his own and related conditions, varies widely according to circumstances; that "what the heart patient"—or any other—"should know," might better be presented to him and to his family by the individual attendant, in accordance with individual and ever-changing conditions.

GOLDEN RULES OF DIAGNOSIS AND TREATMENT, by Henry A. Cables, B. S., M. D., St. Louis; published by the C. V. Mosby Co. of St. Louis as part of the Golden Rules Series; 318 pages; $2.25.

This is the second revised edition, entirely re-written with many additions, especially regarding infectious diseases. The exhaustion of the first edition is a practical expression of the value of the book.

---

THE DIFFICULTIES AND EMERGENCIES OF OBSTETRIC PRACTICE, by Comyns Berkeley, M. A., M. D., M. C., F. R. C. P., M. R. C. S., and Victor Bonney, M. S., M. D., B. Sc., F. R. C. S., M. R. C. P., each of London; published by P. Blakiston's Son & Co., Philadelphia and London; 787 pages, 287 illustrations; $7.50.

We have always held that if new books could omit the fundamental information possessed by every well prepared graduate in medicine, and could be limited to the new, difficult and more highly technical points of a given special field, they would be more easily read and more cheerfully bought. This work fulfills this requirement of economy of time and money almost ideally. At the same time the authors have not taken too much for granted but have sufficiently alluded to general principles underlying the proper conception of difficult and emergent conditions.

---

PROGRESSIVE MEDICINE, June, 1913. Quarterly, $6.00 per year. Edited by Hobart A. Hare and Leighton F. Appleman of Philadelphia; published by Lea & Febiger, Philadelphia.

This number contains the following reviews: Hernia, Wm. B. Coley; Abdominal Surgery, John C. A. Gerster; Gynaecology, John G. Clark; Blood, Diathetic and Metabolic Diseases, Thyroid, Lymphatic System, Alfred Stengel; Ophthalmology, Edward Jackson. The following impress us as especially interesting: Apparatus for treating cystitis with iodine vapor, Farnarier; Artificial Vagina constructed from hernial sac removed from a man, Dreyfus. It may be remembered that last year we suggested that some of the editors were missing the general purpose of this splendid review of literature by introducing what were practically original papers, although these were of the highest order. No such criticism can be made of the present volume. The editors have actually reviewed the literature and have selected the most notable communications.

MULLER'S SERODIAGNOSTIC METHODS. Ross C. Whitman, B. A., M. D., Denver. Authorized translation from third German Edition; 146 pages, 7 illustrations; $1.50. J. B. Lippincott Co., Philadelphia.

Methods of injecting animals, obtaining blood from animals and human beings, preservation of sera, precipitin, floccule, agglutination, bactericide, cytolytic and antihaemolytic reactions are described as a preliminary to the tests for specific diseases. Syphilis, cancer, gonorrhoea, tuberculosis, cobra venom, are then discussed, as well as the antryptic, meiostagmin and phagocytic reactions. Miller's aim has been to make his work practical and, for that reason, the descriptions are full and satisfactory. The translator has caught the spirit of the author but has succeeded in avoiding a too literal translation. The book is an excellent manual, especially for one unfortunately deprived of the opportunity for personal instruction. We venture one minor criticism: Only a very absent-minded reader needs to be told at the head of each page the title of the book which he is reading, but it is a great convenience in turning over the leaves to have the general topic indicated.

---

ELEVENTH ANNUAL REPORT OF THE BUREAU OF SCIENCE (Phillipine Islands) by Alvin J. Cox, Acting Director.

This a copiously illustrated paper-bound book of 78 pages of text, covering the year ending August, 1912. Very wisely, it treats especially of the natural products with reference to their economic value.

---

MUNICIPAL ORDINANCES PERTAINING TO PUBLIC HEALTH, adopted from July 1 to December 31, 1911, by cities of 10,000 population and over. Compiled by John W. Trask, Asst. Surg Gen., U. S. Public Health Service.

It should be understood that this compilation is restricted to new ordinances and rule and is consequently merely supplementary to previous compilations. We note the following rather novel points: San Francisco, limits and boundaries for persons liable by their habits to spread venereal diseases, establishment of clinics; Oil City, restriction of rummage sales; San Francisco, provision of sterilization of rags sold for wiping machinery, etc.; East Providence requires destruction of poison ivy and Jamestown weed; Elgin and Oil City prohibit the distribution of patent medicine samples; Lawrence requires inspection and license of manicuring, massage and vapor bath establishments.

REPORT OF THE N. Y. STATE VETERINARY COLLEGE at Cornell University for the year 1911-12, by the Director, Veranus A. Moore.

This consists largely of scientific articles, including a discussion of abortion and sterility in cattle, tuberculosis of chickens (not pathogenic to rabbits and guinea pigs) and a report of mallein reactions in horses.

---

PICTURE BOOK OF EARLIER BUFFALO. Vol. 16 of the Buffalo Historical Society publications, prepared by Frank H. Severance, Secretary.

This is a most interesting collection of pictures, drawings and photographs, illustrating the history of Buffalo. In April, 1913, Juba Storrs made a map showing the location of all the principal buildings of the village before it was burned by the British. This map covered the area from a little south of Exchange street to a little north of Huron street and from Ellicott to Franklin streets (giving the present names). Dr. Chapin resided on Swan street between Main and Pearl, Dr. Johnson on the west side of Main street nearly as far north as Chippewa. Townsend & Coit, druggists, were located at the southwest corner of Main and Swan, Le Coulteulx, the other druggist, on the west side of Exchange between Main and Washington. The twin pumps on the Terrace, where the writer's uncle went for water, and from which the writer himself drank in the happy days when typhoid was a dispensation of Providence, are shown. The Old Hospital of the Sisters of Charity and the Medical College, the original wing of the General Hospital, are of especial interest to the medical profession. The old brick residences used as city and county buildings before the erection of the present City and County Hall recall some pleasant visits to Dr. Thomas Lothrop, during his term of office as Superintendent of Education.

---

TWENTY-EIGHTH ANNUAL REPORT OF THE BUREAU OF AMERICAN ARCHAEOLOGY, by W. H. Holmes, Chief, published 1912.

This includes an exhaustive description of Casa Grande, Arizona, by Jesse Walter Fewkes; Antiquities of the upper Verde and Walnut Creek Valleys, Arizona, by the same; and a preliminary report on the Linguistic Classification of the Algonquinian Tribes by Truman Michelson. We recommend this last article especially to those who may have the impression that the Indian languages were crude and simple.

Diseases of the Rectum and Pelvic Colon, Martin L. Bodkin, M. D., Brooklyn. E. B. Treat & Co., New York; 416 pages. 90 illustrations from drawings by Francis A. Deck; $3.50.

This is an excellent, sytematic text book, including a brief anatomic and physiologic chapter, one on methods of examination and gradually leading up to the discussion of the various diseased conditions encountered. Medical and surgical measures are well balanced in the therapeutics. The author is conservative, as, for instance, in his caution against using a sigmoidoscope without an obturator, as often advised, in his very fair discussion of the merits and demerits of various specula and of the various operations for haemorrhoids. We personally agree with his preference for the left lateral position for sigmoidoscopy.

---

Laboratory Methods, with Special Reference to the Needs of the General Practitioner, by Drs. B. G. R. and E. G. C. Williams of Traverse City, Mich. The C. V. Mosby Co., St. Louis; 210 pages, 43 engravings; $2.50.

The great demand for this work has exhausted the first edition, and the present, second edition, though only slightly larger, contains a considerable number of additions, simplicity and brevity being the guiding principles of the authors. Dr. H. R. Harrower's apparatus for quickly determining urinary acidity, the albumin sputum test for tuberculosis, Bass & Watkins's Rapid Widal test, Noguchi's butyric acid test for syphilis and the urobilinogin test of hepatic function are among the additions. While designed especially for the general practitioner, the book abounds with ingenious devices and practical hints well worth the attention of even the expert.

---

Blood-Pressure, from the Clinical Standpoint, by Francis Ashley Faught, M. D., of the Medico-Chirurgical College, Philadelphia. Octavo of 281 pages, illustrated; Philadelphia and London, W. B. Saunders Co., 1913; price, $3.00 net.

Approximately a quarter of the work deals with physiologic principles, the description of types of instruments, general methods of use, standards, etc. There follows a systematic consideration of the significance of blood pressure in different diseases, surgical, obstetric and insurance practice, and the last forty pages are devoted to a thorough consideration of the methods of controlling blood pressure.

ACUTE INTESTINAL INFECTIONS OF CHILDREN. This an interesting pamphlet prepared by the Lambert Pharmacal Co. of St. Louis.

---

THE DOCTOR'S FACTOTUM. The July issue of this journal, published by the N. Y. Pharmacal Assn., Arlington Chemical Co. and Palisade Mfg. Co. of Yonkers, should have been received by every physician in the country. If any has failed to receive it he should send his address, as it contains not only the brightest medical wit and humor published but much valuable information. Publications of the general nature of the last two mentioned should not be confused with the ordinary run of circulars.

---

THE PHYSIOGRAPHY OF THE RIO GRANDE VALLEY, N. M., in Relation to Pueblo Culture, by E. L. Hewitt, Junius Henderson and W. W. Robbins. Bureau of American Ethnology, Bulletin No. 54.

This is the story, told in scientific terms, from the evidence of geology and achaeology, of the gradual depopulation of a once comparatively fertile territory. Climatic changes, with diminishing water supply, in spite of an extensive system of irrigation, made the conditions of life harder and harder till the very considerable cliff dwellers were finally quite abandoned, probably a thousand years ago.

---

STERILITY IN THE MALE AND FEMALE AND ITS TREATMENT. Max Huhner, M. D., New York, published by the Rebman Co., New York; 262 pages; cloth; $2.00.

The author points out that, in spite of various lesions, active spermatozoa may be present and, on the other hand, that, even if present, there may be male abnormalities or functional disorders which prevent their proper deposit. He, therefore, holds that the true test of male or female sterility, depends upon whether living spermatozoa can be removed from the cervix after coitus. The work is not only a thorough, analytic treatise on the subject, but enters into the physiology of impregnation. Its careful reading will dispel many erroneous generalizations, tend to prevent haphazard treatment and result not only in better professional knowledge but, ultimately, will have an influence on serious sociologic problems.

SEX, ITS ORIGIN AND DETERMINATION, Thomas E. Reed, M. D., Middletown, O.; published by the Rebman Co., New York; 313 pages; cloth; $3.00.

In view of the general skepticism as to the possibility of determining sex in advance of birth, either in the etiologic or diagnostic sense, and the difficulty of doing justice either to the author's or other theories of the influence due to food, metabolic cycles, etc., in a brief review, we prefer not to anticipate the reader's attention to this book. It is a thoughtful and elaborate treatise and deserves the attention of those especially interested in sociologic problems.

---

THERAPEUTICS OF THE GASTRO-INTESTINAL TRACT, authorized translation of Dr. Carl Wegele's work in German, by Drs. Maurice H. Gross and I. W. Held of New York. Published by the Rebman Co., New York; 329 pages; cloth; $3.00; illustrated.

The translators have adapted the original work by condensation in parts and by important additions, as the consideration of the duodenal tube. The work is much broader than its title, entering into diagnosis and other matters so as to base therapeutic suggestions on a proper understanding of the subject.

---

THE PRACTICAL MEDICINE SERIES, Vol 1, General Medicine, edited by Frank Billings, M. S. M. D., and J. H. Salisbury, A. M., M. D., of Chicago. Vol. 2, General Surgery, edited by John B. Murphy, A. M., M. D., LL. D., Chicago. The series under the general editorship of Gustavus P. Head, M. D., and Charles L. Mix A. M., M. D., of Chicago.

The yearly series consists of ten volumes, price $10. The price of Vol. 1 (404 pages) is $1.50, and of Vol. 2 (632 pages) $2.00. All of the volumes are abundantly illustrated and each consists of a review of the literature of the branch covered by the special volumes, up to date of publication and covering approximately the year. This series is the best extant review of medical literature.

---

DIGEST OF LAWS AND REGULATIONS IN FORCE IN THE U. S., relating to the possession, use, sale and manufacture of poisons and habit-forming drugs, by M. I. Wilbert and Murray Galt Motter. U. S. Public Health Service Bulletin No. 56.

SUMMARIES OF LAWS RELATING TO THE COMMITMENT AND CARE OF THE INSANE IN THE U. S., prepared by John Koren for the National Committee for Mental Hygiene.

The value of such a compilation, both for reference and to secure the best possible legislation, needs no comment.

---

TEXT BOOK OF DISEASES OF THE NOSE, THROAT AND EAR, Francis R. Packard, M. D., Philadelphia, published by the J. B. Lippincott Co., Philadelphia and London; 377 large pages; 145 illustrations; $3.50.

This volume is uniform with others of the New Medical Series and is a complete, systematic treatise of the specialty.

---

MASSAGE. Dr. Douglas Graham of Boston, with a chapter on Massage of the Eye by Dr. A. Darier of Paris, published by the J. B. Lippincott Co., Philadelphia and London; 574 pages; 75 illustrations; fourth edition, revised and enlarged.

The book begins with a history of massage and, indeed, throughout, it is replete with references to the early literature. Possibly some critics may object to the number of references to journals published one to three decades ago, too old to be considered modern, too recent to be historic. The book is very readable aside from its practical and scientific value as the author, without ever losing sight of his purpose, is full of dry wit, from the title page, where he quotes Job xxxi, 35: "Oh that mine adversary had written a book!" to the end.

---

GONORRHEA IN WOMEN, Its Pathology, Symptomatology, Diagnosis and Treatment; together with a review of the rare varieties of the disease which occur in men, women and children. By Charles C. Norris, M. D., Instructor in Gynecology at the University of Pennsylvania. Octavo of 521 pages, illustrated; Philadelphia and London, W. B. Saunders Co., 1913; cloth, $6.00 net; half morocco, $7.50 net.

An interesting historic chapter opens the work. Bacteriology and pathology are then discussed. Non-genital lesions are treated in a very thorough manner. The chapter on therapeutics is valuable, especially on account of the adoption of the Missourian method of presenting bacteriologic tables showing the effects of various antiseptics in strengths ordinarily used. The size of the work is due, not to literary effusiveness, but to the thoroughness and completeness with which the author has conceived his task.

Vaccine and Serum Therapy, including a Study of Infections, Theories of Immunity, Specific Diagnosis and Chemotherapy, Edwin Henry Schorer, B. S., M. D., Dr. P. H., Kansas City. Published by the C. V. Mosby Co., St. Louis; 300 pages; $3.00

This is a systematic treatise, both theoretic and practical, covering the ground as fully as possible in the present state of our knowledge, and not marked by undue enthusiasm over untried methods.

---

Medical Union Number Six, Wm. Harvey King; 60 pages; cloth; Boericke & Tafel, Philadelphia; 50 cents.

This little story applies to our own profession, doubtless in a somewhat exaggerated degree, the characteristics of a trade union. It is a story with a purpose and we wish it might be read by every working man—meaning by working man those who work only eight hours a day and five and a half days a week, when allowed to do so by professional laborers who ride in Pullman cars and put up at high-price hotels.

---

Collected Papers By the Staff of St. Mary's Hospital (Mayo Clinic) for 1912. Octavo of 842 pages, 219 illustrations; Philadelphia and London, W. B. Saunders Co., 1913; cloth, $5.50 net.

The large number of conditions treated renders it impracticable to review this work in detail. The papers are concise, even when of considerable length and evince throughout the most careful study, wide experience and a judicial attitude based on actual statistics, well digested.

---

Diseases of the Eye. By George E. deSchweinitz, M. D., Professor of Ophthalmology in the University of Pennsylvania. Seventh edition, thoroughly revised. Octavo of 979 pages, 360 text illustrations and seven lithographic plates; Philadelphia and London, W. B. Saunders Co., 1913; cloth, $5 net; half morocco, $6 net.

It was the privilege of the reviewer to become acquainted with deSchweinitz's work soon after he had emerged from the preparatory stage essential to every practitioner. His text book, appearing first in 1892, was already the expression of large experience and matured thought. Both the man and the book have grown with the years, and it is necessary for us merely to call attention to the number of the edition, the completeness of the work and the fact that it has been accepted as standard authority for more than twenty years.

---

THE SURGICAL CLINICS OF JOHN B. MURPHY, M. D., at Mercy Hospital, Chicago. Volume II, Number 3 (June, 1913). Octavo of 185 pages, 62 illustrations; Philadelphia and London, W. B. Saunders Co., 1913; published bi-monthly; price, per year, paper, $8.00; cloth, $12.00.

This number contains the most varied collection of subjects and less than one-third have anything to do with either bones or joints. In fact, one subject connected under this heading belongs very distinctly to Orthopaedic surgery, though the method of bone-grafting seems to make this distinction old-fashioned. We refer to Dr. F. H. Albee's operation of bone-grafting for the cure of Potts Disease. In the introduction Dr. Murphy points out that by such operations orthopaedic surgery has become not a borderline surgery but a part of real surgery. Some twenty-two pages are devoted to this clinic. Another subject usually considered under Gynaecology is Dr. Murphy's method of treating a Procidentia Uteri. From Genito-Urinary Surgery we note a case of acute Suppurative Prostatitis, which is treated by drainage. On the whole the volume is very inspiring as illustrating the kind of work done by an up-to-date active operator.

